# Who Is Classified as Untestable on Brief Cognitive Screens in an Acute Stroke Setting?

**DOI:** 10.3390/diagnostics9030095

**Published:** 2019-08-14

**Authors:** Emma Elliott, Bogna A. Drozdowska, Martin Taylor-Rowan, Robert C. Shaw, Gillian Cuthbertson, Terence J. Quinn

**Affiliations:** Institute of Cardiovascular and Medical Sciences, University of Glasgow, New Lister Building, Glasgow Royal Infirmary, Glasgow G31 2ER, UK

**Keywords:** feasibility, cognitive screening instruments, cognition, stroke

## Abstract

Full completion of cognitive screening tests can be problematic in the context of a stroke. Our aim was to examine the completion of various brief cognitive screens and explore reasons for untestability. Data were collected from consecutive stroke admissions (May 2016–August 2018). The cognitive assessment was attempted during the first week of admission. Patients were classified as partially untestable (≥1 test item was incomplete) and fully untestable (where assessment was not attempted, and/or no questions answered). We assessed univariate and multivariate associations of test completion with: age (years), sex, stroke severity (National Institutes of Health Stroke Scale (NIHSS)), stroke classification, pre-morbid disability (modified Rankin Scale (mRS)), previous stroke and previous dementia diagnosis. Of 703 patients admitted (mean age: 69.4), 119 (17%) were classified as fully untestable and 58 (8%) were partially untestable. The 4A-test had 100% completion and the clock-draw task had the lowest completion (533/703, 76%). Independent associations with fully untestable status had a higher NIHSS score (odds ratio (OR): 1.18, 95% CI: 1.11–1.26), higher pre-morbid mRS (OR: 1.28, 95% CI: 1.02–1.60) and pre-stroke dementia (OR: 3.35, 95% CI: 1.53–7.32). Overall, a quarter of patients were classified as untestable on the cognitive assessment, with test incompletion related to stroke and non-stroke factors. Clinicians and researchers would benefit from guidance on how to make the best use of incomplete test data.

## 1. Introduction

Cognitive screening following a stroke is recommended in international clinical guidelines [1,2] and routinely performed in acute stroke settings in many countries. However, completion of a cognitive test battery in a medically unwell person with recent neurological insult is challenging. Previous research has demonstrated that around 20% of stroke patients cannot fully complete many of the cognitive screening tests commonly used in stroke practice, for example the Montreal Cognitive Assessment (MoCA) [3] and the Mini-Mental State Examination (MMSE) [4]. Test non-completion is reported in both acute stroke [5] and rehabilitation settings [6] (Table 1). However, published data appear conflicting and other centres have reported that a lengthy neuropsychological battery can be performed in the acute setting [7].

Feasibility of completing a cognitive assessment is multifactorial; some aspects may relate to the stroke (extent of damage, presence of aphasia, limb weakness) and others may relate to the nature of the testing (timing and length of assessment, complexity). Looking at the patient characteristics and approaches to assessment can explain the apparently contradictory findings in the literature. Patients included in studies of cognitive tests are often not representative of a typical stroke unit. For example, studies may favour the inclusion of those with minor strokes, no (or little) pre-stroke disability and those who are able to provide informed consent, whilst patients with severe aphasia or an existing diagnosis of dementia are often excluded [17]. This selection bias will underestimate the true incidence of untestable patients.

An incomplete cognitive test has clinical implications. Inexperienced assessors may erroneously ascribe an incomplete test to cognitive impairment, when in the context of stroke, non-completion may relate to physical impairments. Ultimately, test non-completion could risk false positive and false negative diagnosis of cognitive problems with attendant harm. An understanding of the extent of test non-completion and knowledge of factors relating to untestability could potentially avoid this. The issue of test incompletion also complicates stroke research and audit. Often patients with incomplete assessments are excluded from analyses (since a total score cannot be calculated). This practice biases results, underestimates levels of cognitive impairment and could also lead to erroneous results [11]. Various approaches to incorporate incomplete tests have been proposed but there is no consensus on the best method [16].

There are different ways to address these feasibility issues, but the approach taken will depend on the aspect of feasibility of greatest relevance. For example, one may decide to choose a test specifically designed for a stroke (e.g., Oxford Cognitive Screen (OCS) [18]). This approach recognises that many traditional cognitive tests were designed for memory clinic populations and are not suited to the specific challenges encountered in acute stroke settings. Stroke specific, multi-domain tests are described and may be less biased by physical, communication and visuospatial impairments. Another approach may be to choose a shorter cognitive screen. This approach may be particularly suited to the acute medical setting where clinicians have limited time and other investigations may be prioritised in the first few days. Shorter tests may also be attractive to patients as there will be a reduced test burden. Short cognitive screens have been largely ignored in research conducted in the stroke setting. Stroke care is continuously evolving and differs internationally, but there is currently a paucity of feasibility research on cognitive tests in an acute, National Health Service (NHS) context. Our research aimed to meet these two gaps.

Our primary aim was to describe the test completion (feasibility) of some of the shortest cognitive screens (deliverable in under five min) in an unselected group admitted to our hyper-acute stroke unit. Our secondary aims were to explore reasons for assessors giving a patient a label of being untestable and to describe factors associated with being untestable.

## 2. Methods

We conducted an observational, cross-sectional study, using routinely collected data from an urban UK, teaching hospital. This was approved by the West of Scotland Research Ethics Committee (ws/16/0001) on 4 February 2016. We followed Standards of Reporting of Neurological Disorders (STROND) guidance [19] for the design, conduct and reporting of the study.

### 2.1. Setting and Population

We collected anonymised, routine, clinical data from consecutive admissions to our hyper-acute stroke unit (HASU). The unit admits all suspected stroke and transient ischaemic attack (TIA) patients with no exclusions in relation to age, disability or comorbidity. The unit offers level two (high dependency) clinical care and only patients requiring multi-organ support would be admitted to a higher-level care facility. Recruitment occurred during four timepoints: May 2016–February 2017; April–June 2017; October–December 2017; and July–August 2018. For the purposes of this study, we made no exclusions around stroke severity or stroke-related impairments. Written informed consent was not required for assessment.

### 2.2. Clinical and Demographic Assessment

Clinical and demographic data were collected for each patient by five trained researchers (four postgraduate students in psychology/neuroscience and one undergraduate medical student). Data collected were a mix of prospective assessment and retrospective derivation from medical case notes. Stroke severity was determined by the National Institutes of Health Stroke Scale (NIHSS) [20] on admission. Medical history, including any pre-stroke diagnosis of dementia, was recorded using medical notes and primary care summary data. Pre-stroke functioning was established using the modified Rankin Scale (mRS) [21,22,23]. Bamford stroke classification was completed for both ischaemic and haemorrhagic patients.

### 2.3. Cognitive Assessment

The cognitive assessment consisted of 13 questions, covering 8 different cognitive screening tests: the 10-point Abbreviated mental test score (AMTS) [24] and its shorter version AMT-4 [25], General Practitioner Assessment of Cognition (GPCOG) (patient section) [26], Mini-Cog [27], six item cognitive impairment test (6-CIT) [28], National Institute Neurological Disorders S-Canadian Stroke Network (NINDS-CSN) 5-min MoCA [29], abbreviated MoCA [30], and the 4 ‘A’s Test (4AT) (Available online: www.the4AT.com) (Table 2). Each of these individual tests can be administered in under 5 min (and so suitable for use in acute clinical practice). They cover a variety of cognitive domains and have some supporting validation work in primary and geriatric care [31].

Assessment was attempted during the first week of admission. Patients were only approached once for assessment, unless the patient requested for the assessment to be done at a later time-point, the assessment was interrupted by another clinical investigation (e.g., scan) or if the patient requested the assessment to be done over two sessions. Patients were not approached at all (and categorised fully untestable) if the parent clinical team reported that the patient was too unwell to undergo assessment or if the assessor felt that any form of direct testing would not be possible. In these patients, who could not be directly assessed, we checked if a cognitive assessment was documented by the parent clinical team since admission.

### 2.4. Defining the Test Completion Outcomes

Patients were classified as fully untestable when no part of the assessment was attempted (decision made by researcher in consultation with parent clinical team) and/or when no questions were answered when testing was attempted. Partially untestable was defined when at least one item in a test could not be completed or was not attempted (decided by either the patient, parent clinical team or researcher). A list of potential categories was created by the authors based on clinical experience, previous literature and initial scoping of free text responses. The free text reasons documented for each patient classified as untestable by the individual assessor were later collated into categories (e.g., aphasia and dysarthria both captured under speech problems) by the lead author (E.Elliott) with discussion with the stroke consultant (T.Quinn). Where more than one reason was listed, we chose the primary factor deemed to have the greatest impact on assessment (e.g., a patient documented as both acutely confused and dysarthric was categorised under confusion). Cases where a test item was attempted but poorly completed, for example, a patient with limb weakness who attempted the clock-draw with their weak or non-dominant hand, were classed as testable.

### 2.5. Statistical Analysis

We described patients as fully or partially untestable using the definitions above. We looked at the completion rate of each question in our assessment and then calculated completion rates for the different tests. Patients who ended up with a non-stroke diagnosis were kept in the analyses as we were interested in the feasibility of tests within all patients admitted with a suspected stroke and we retained admission NIHSS for these patients where it was completed.

We assessed univariable and multivariable associations with outcomes of interest using logistic regression. Variables were chosen based on previous literature [10,11] and plausible associations with feasibility. The following 12 covariates were used in both univariate and multivariate analyses: age (years), sex, NIHSS, Bamford stroke classification (TIA, partial anterior circulation stroke—PACS, total anterior circulation stroke—TACS, posterior circulation stroke—POCS, lacunar stroke—LACS, non-stroke (used as reference group)), pre-morbid mRS, presence of intracerebral haemorrhage (ICH), previous diagnosis of dementia and previous TIA/stroke. We did not include delirium in the model since our only measure was the 4AT scale and all untestable patients would have ended up with a label of delirium. Associations were described as odds ratios (OR) with corresponding 95% confidence intervals. We used the rule of 10 outcome events per predictor variable to determine the number of covariates we could include in the model and so required 120 “cases” for the model. 

Analyses were run twice to account for how partially untestable patients are treated differently in the literature; in the first analysis they were treated as testable and in the second treated as untestable (grouped with the fully untestable patients). All data analyses were performed using the statistical software package SPSS (version 25 IBM, Armonk, NY, USA).

## 3. Results

**Figure 1:**
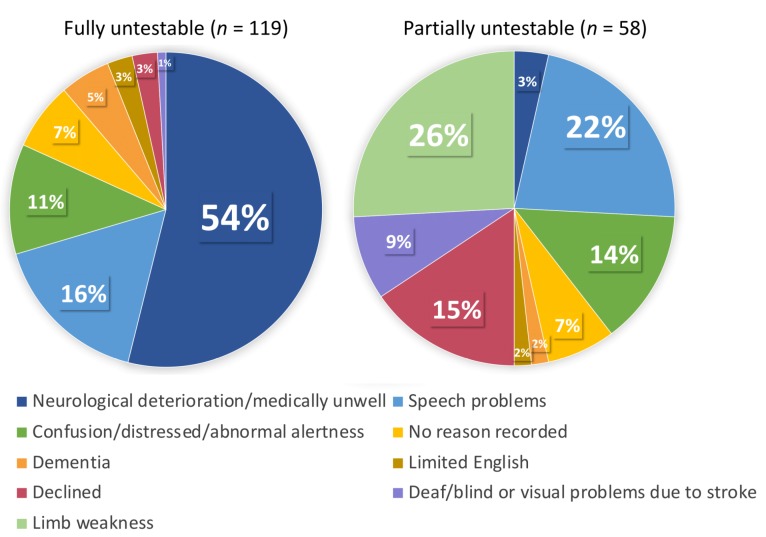
Reasons for fully/partially untestable.

The full sample included 703 patients (mean age 69.4 ± 13.7, 382 (54%) males, median NIHSS 2 (interquartile range, IQR 1–5)) (Table 3 for full patient characteristics). Of these, 119 (17%) were classified as fully untestable on all tests. Reasons for fully untestable fell under eight categories but for more than half of the group this was due to neurological deterioration (e.g., patients who were unresponsive, very unwell, palliative) (62/119, 54%). A further 58 (8%) patients in the full sample were separately classified as partially untestable (did not attempt ≥1 question); reasons fell under nine categories, with limb weakness 15 (26%) and speech problems 13 (22%) being most prevalent (full breakdown of reasons detailed in Figure 1). A large proportion of patients in the fully untestable group (*n* = 50, 42%) had a TACS, compared to only 3 (5%) in the partially untestable group.

Patients who ended up with a non-stroke diagnosis (*n* = 109) were a diverse group (diagnoses included migraine, subarachnoid haemorrhage and vasovagal events). Of these, 20 (18%) were untestable in some way. Only 12 patients (2%) of the full sample declined the cognitive assessment; three declined the full assessment and nine declined certain questions. Characteristics: 7 (58%) males, mean age of 74.3 (SD = 13.9), median NIHSS of 3 (IQR 2–5), diagnoses: 1 non-stroke, 3 TIAs, 3 PACS, 3 POCS and 2 LACS.

We looked at the completion of each individual question within our full cognitive assessment (Table S1). Clock-draw had the lowest completion rate (533/703 (76%)), whilst age had the highest (583/703 (83%)). For 25/58 (43%) patients in the partially untestable group, clock-draw was the only task that they did not attempt. The completion rate of each individual test is given in Table 2; the 4AT was the only test which could be scored in full for all patients.

In the univariate analyses: higher age, TACS, ICH, higher NIHSS, higher pre-morbid mRS and a previous diagnosis of dementia were associated with being untestable, whilst a lacunar stroke was associated with being testable (Table 4). In the first multivariable regression analysis (*n* = 680), independent associations with fully untestable status were: higher NIHSS score (OR: 1.18, 95% CI: 1.11–1.26), higher pre-morbid mRS (OR: 1.28, 95% CI: 1.02–1.60) and pre-stroke dementia (OR: 3.35, 95% CI: 1.53–7.32). A lacunar stroke classification was associated with being testable (OR: 0.19, 95% CI: 0.06–0.65). In the second analysis (where the partially untestable group was combined with the fully untestable), the above variables remained significant. In addition, the following associations were found for being untestable: older age (OR: 1.04, 95% CI: 1.02–1.06) and presence of ICH (OR: 3.44, 95% CI: 1.13–10.44); whilst a TIA classification was associated with being testable (OR: 0.45, 95% CI: 0.20–0.997) (Table 4).

## 4. Discussion

In an unselected sample of 703 patients admitted to our HASU, a quarter were classified as partially or fully untestable on brief cognitive screening tests. In those patients classified as partially untestable, the clock-draw was the most problematic, so tests including this item had the lowest completion rate. The 4AT was the only test which could be scored in full for all patients as it includes a score for being untestable. Factors associated with being fully untestable were previous diagnosis of dementia, higher pre-morbid mRS and higher NIHSS on admission, whilst a diagnosis of lacunar stroke was associated with being testable.

### 4.1. Research in Context

Our findings are generally in keeping with the limited literature on test feasibility. The associations of non-completion with stroke severity and dementia have face validity and the reasons given for a label of untestable were similar to those described in previous studies (for example limb weakness [5,11,16], aphasia [5,10,11], pre-morbid functional status [5] and reduced consciousness [11]), although reporting reasons for cognitive test non-completion in research is the exception rather than the norm. These findings highlight that non-completion is driven by both stroke specific and non-stroke related factors. Our finding that the clock-draw was the most problematic test is also in keeping with previous research findings for a stroke population; Lees et al. [16] found the lowest rates of completion on test items that required copying or drawing. Although tasks which assess visuospatial abilities, such as the clock-drawing test, can be challenging for stroke patients, they provide useful information on a key cognitive domain and can predict longer-term outcomes [32].

We decided to focus on the shortest cognitive tests available, in the hopes that they would be more practical for both the patient and clinician. Our results showed that the rates of completion for these short tests were similar to the completion rates for longer multi-domain cognitive tests previously studied (MoCA, MMSE). This should not be interpreted as meaning that the shortest tests are just as likely to be incomplete as more detailed tests. We did not include or directly compare longer tests with our short screens and our unselected population is not comparable with the patients tested in previous studies. There is a concern that shorter cognitive tests are inferior to longer, more detailed tests. Previous work has suggested that there is a trade-off between duration of administration and diagnostic accuracy [33] in the context of dementia. A focus on length of assessment alone (number of questions, administration time) is perhaps too simplistic, and test content is likely to be more important. For example, a long test could assess one area of cognition in depth yet neglect other domains.

### 4.2. Strengths and Weaknesses of the Research

A major strength of our study is that we had access to an unbiased, real-world sample, including patients who are often excluded from research (for example those with severe aphasia and dementia). While using clinical data have these benefits, we also have to acknowledge that due to the ‘messy reality’ of acute clinical practice, data are often missing. Our approach allowed us to retrospectively derive missing data from various sources including inpatient medical records, primary care data and consultation with the parent clinical team. Retrospective scoring can increase some inaccuracy, for example, calculating NIHSS based on the symptoms documented in medical case notes, rather than carrying it out directly with the patient.

There were some potentially interesting aspects of feasibility/applicability where we did not record data. We did not record the total number of patients who had limb weakness from their stroke and attempted the clock-draw using their weak or non-dominant hand (classed as testable). Data on this subgroup would be useful as many will lose points or score zero for poorly completed drawing tasks. We also did not record if an assessment had to be completed over two sessions or if any part of the assessment was interrupted.

Although we operationalised our concept of partially and fully untestable there is still subjectivity in the interpretation. It is essentially a judgement call by the clinician whether patients with aphasia, limb weakness and visual problems can complete a task (if the patient does not decline themselves). The same patient could therefore be classified differently purely based on who assessed them. This is particularly relevant in our study, where differing assessors performed the cognitive testing. This could be considered both a strength and weakness as it provides further real-world validity (some people might be better at encouraging patients to complete an assessment than others).

Finally, a limitation of determining feasibility of different questions and their resulting tests is the order in which the questions are asked. We acknowledge asking questions in the same order for each patient introduces some bias and is an issue because some patients will struggle to focus for longer periods of time or are easily fatigued.

### 4.3. Recommendations for Future Research and Practice

The strict administration and scoring criteria required for cognitive tests can be problematic for the stroke setting. Clinicians and researchers can therefore expect to encounter a number of stroke patients that will be untestable on certain tasks, or patients who are testable, but their stroke-related impairments result in a misleading test score. While in clinical practice an assessment can be put into context, in research it is more important that a-priori rules are set for dealing with incomplete tests. The importance of doing this is highlighted by the fact our analyses showed different results depending on how partially untestable patients were classified. Numerous approaches exist to deal with missing data [16], but to maximise the utility of the data collected, we recommend, where possible, that researchers make full use of incomplete participant data, rather than applying a complete-case analysis approach.

Tests which incorporate scoring for untestable patients, such as the 4AT, are helpful. Although the 4AT is primarily a delirium screen, the same approach could be applied to general cognitive tests. Guidance documents exist for scoring other stroke scales such as the NIHSS in patients who are comatose, confused, etc., so these types of resources could be made available for challenging cases in cognitive assessment.

**Figure 2:**
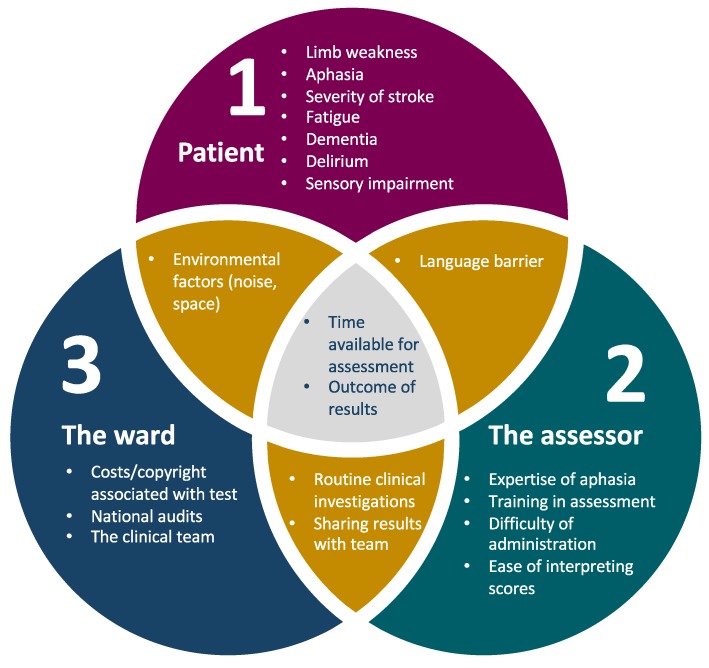
Factors affecting feasibility of cognitive assessment in acute stroke. Factors listed are illustrative but not exhaustive.

Test completion rates are just one measure of feasibility. Feasibility covers a range of factors relating to the patient, assessor and the ward setting (Figure 2), so future research studies should include data addressing these other perspectives. To date, there has been little data published on the clinician’s experience of cognitive assessment, environmental factors affecting assessment on the ward (noise, space, interruptions) and practical aspects, such as how assessors have misinterpreted administration/scoring instructions. With the increased use of computerised versions of cognitive tests in the future, feasibility issues from the assessor’s side are likely to improve; for example, automatic scoring saves time and reduces scoring errors and subjectivity. Future research should also make use of routinely collected clinical data, such as that collected by the Sentinel Stroke National Audit Programme (SSNAP) and the Scottish stroke care audit in the United Kingdom. One could argue that any study using a researcher to administer a scale, rather than a clinical member of staff, is not truly addressing broader feasibility and implementation issues.

## 5. Conclusions

In a real-world sample, a quarter of patients in our HASU were classified as fully or partially untestable on brief cognitive screening tests. Clinicians and researchers should make a-priori plans on how to address incomplete assessments. Feasibility is a multi-faceted term, and factors from both clinician and patient point of view should be considered.

## Figures and Tables

**Table 1:** Previous studies addressing feasibility of cognitive assessments post-stroke.

Study	Test	Number of Patients	Inclusion Criteria Relevant to Feasibility	Time Point	Completion Rate
**Setting: Acute**					
Alderman et al. [8] (CA)	Battery of 8 tests	27	Mild strokes and TIAs	≤24 h	96%
Collas 2016 [9] (CA)	OCS	155	No relevant exclusions	5 days (mean)	89%
Horstmann et al. [10]	MoCA	842	IS and ICH. No relevant exclusions	2 days (median)	81%
Pasi et al. [5]	MoCA	137	IS and ICH. No relevant exclusions	5–9 days	83%
Pendlebury et al. [11]	AMT MMSE	1097	No relevant exclusions	4 days (median)	76% partially testable 69% fully testable
Van Zandvoort et al. [7]	1.5-h NPB	57	IS only, no previous stroke, maximum age 80, mRS 2–4, no psychiatric history or comorbidity that could influence cognitive functioning	4–22 days	75%
**Setting: Sub-Acute/Rehabilitation**					
Barnay et al. [12]	CASP MMSE MoCA	44	All aphasic patients	42 ± 22 days	CASP 82% MMSE 64% MoCA 70%
Benaim et al. [6]	CASP MMSE MoCA	50	Non-aphasic patients only	40 ± 17 days	CASP 100% MMSE 100% MoCA 94%
Cumming et al. [13]	MoCA	220	IS and ICH. No relevant exclusions	3 months	Mild stroke 87% Moderate stroke 79% Severe stroke 67%
Kwa et al. [14]	CAMCOG	129	IS only	≥3 months	88%
Mancuso et al. [15]	OCS MMSE	325	No previous stroke, able to consent themselves, no previous psychiatric/neurological disease	33.9 ± 41.8 days	Fully untestable: MMSE 2%, OCS 1% Highest incompletion for individual OCS tasks: trails 28/325 (9%)
Lees et al. [16]	ACE-III MMSE MoCA	51	No relevant exclusions	36 days (median)	ACE-III 27% MMSE 43% MoCA 39%

Abbreviations: Abbreviated Mental Test (AMT); Addenbrooke’s Cognitive Examination III (ACE-III); Cambridge Cognition Examination (CAMCOG); Cognitive assessment scale for stroke patients (CASP); conference abstract (CA); ischaemic stroke (IS); intracerebral haemorrhage (ICH); Mini-Mental State Examination (MMSE); Montreal Cognitive Assessment (MoCA); neuropsychological battery (NPB); Oxford Cognitive Screen (OCS); transient ischaemic attack (TIA); modified Rankin Scale (mRS).

**Table 2:** Short cognitive tests ordered by number of items.

Test Name	Number of Items	Questions	Maximum Score	% of Assessments We Could Score in Full
Mini-Cog [27]	2	1 3-word delayed recall 2 Clock draw (numbers, hands)	5	75%
Abbreviated MoCA [30]	2	1 5-word delayed recall 2 Clock draw (face, numbers, hands)	8	75%
4-AMT [25]	4	1 Age 2 Year 3 Place 4 Date of birth	4	81%
4AT (Available online: www.the4AT.com)	5	1 Alertness 2 Age 3 Current year 4 Place 5 Date of birth 6 Months backwards	12	100%
6-CIT [28]	6	1 Time 2 Month 3 Yearl 4 Count backwards from 20 5 5-part delayed recall 6 Months backwards	28	78%
GPCOG [26]	7	1 Date 2 Month 3 Year 4 Date of birth 5 5-part delayed recall 6 Clock draw (numbers, hands) 7 News item	9	75%
NINDS-CSN 5 min MoCA [29]	7	1 Date 2 Month 3 Year 4 Day 5 Place 6 City 7 5-word delayed recall 8 Fluency (letter F)	12	79%
10-AMT [24]	10	1 Age 2 Time 3 Year 4 Place 5 Two-person recognition 6 Date of birth 7 Year of WW1 8 Current prime minister 9 Count backwards from 20 10 3-part delayed recall	10	79%

Abbreviations: Abbreviated mental test (AMT); General Practitioner Assessment of Cognition (GPCOG); Montreal Cognitive Assessment (MoCA); National Institute of Neurological disorders and stroke and the Canadian stroke network (NINDS-CSN); Six item cognitive impairment test (6-CIT).

**Table 3:** Characteristics of the sample.

Characteristics	Full Sample (*n* = 703)	Partially Untestable (*n* = 58)	Fully Untestable (*n* = 119)
Sex (male)	382 (54%)	27 (47%)	59 (50%)
Age mean (SD)	69.4 (13.7) Missing data (*n* = 2)	76.6 (9.7)	76.8 (12.5) Missing data (*n* = 2)
IS ICH TIA Non-stroke	429 IS 22 ICH 137 TIA 109 N/S Missing data (*n* = 6)	42 ΙS 4 ΙCH 5 ΤΙA 7 Ν/S	85 IS 8 ICH 11 TIA 13 N/S Missing data (*n* = 2)
Bamford classification (IS and ICH)	66 TACS 174 PACS 100 POCS 111 LACS Missing data (*n* = 6)	3 TACS 25 PACS 12 POCS 6 LACS	50 TACS 31 PACS 8 POCS 4 LACS Missing data (*n* = 2)
NIHSS median (IQR)	2 (1–5) Missing data (*n* = 15)	4 (3–7) Missing data (*n* = 1)	8 (4–16) Missing data (*n* = 2)
Pre-morbid mRS median (IQR)	1 (0–3) Missing data (*n* = 5)	2 (0–3)	3 (0–3) Missing data (*n* = 1)
Previous stroke (IS/ICH) or TIA (yes)	218 (31%)	20 (34%)	36 (30%)
Previous diagnosis of dementia (yes)	61 (9%)	8 (14%)	30 (25%)

Abbreviations: ischaemic stroke (IS); interquartile range (IQR); intracerebral haemorrhage (ICH); lacunar stroke (LACS); modified Rankin Scale (mRS); National Institute for Health Stroke Scale (NIHSS); non-stroke (N/S); partial anterior circulation stroke (PACS); posterior circulation stroke (POCS); transient ischaemic attack (TIA); total anterior circulation stroke (TACS).

**Table 4:** Feasibility associations.

Variables	Univariate for Fully Untestable	Multivariate (Partially Treated as Testable)	Multivariate (Partially Treated as Untestable)
	**OR (95% CI)**	**OR (95% CI)**	**OR (95% CI)**
Age (years)	**1.06 (1.04–1.08)**	1.02 (1.00–1.04)	**1.04 (1.02–1.06)**
Sex (male)	0.80 (0.54–1.18)	1.32 (0.77–2.26)	0.97 (0.62–1.51)
Stroke classification (non-stroke used as reference group):			
*TACS*	**23.08 (10.29–51.76)**	2.96 (0.98–8.93)	1.47 (0.50–4.34)
*PACS*	1.60 (0.80–3.22)	0.73 (0.32–1.65)	0.92 (0.46–1.83)
*LACS*	**0.28 (0.08–0.88)**	**0.19 (0.06–0.65)**	**0.26 (0.10–0.64)**
*POCS*	0.64 (0.25–1.62)	0.39 (0.14–1.12)	0.73 (0.33–1.61)
*TIA*	0.65 (0.28–1.50)	0.55 (0.21–1.40)	0.45 (0.20–1.00 *)
ICH	**2.96 (1.21–7.23)**	2.48 (0.72–8.59)	**3.44 (1.13–10.44)**
NIHSS	**1.30 (1.23–1.36)**	**1.18 (1.11–1.26)**	**1.23 (1.14–1.31)**
Pre-morbid mRS	**1.64 (1.41–1.91)**	**1.28 (1.02–1.60)**	**1.24 (1.03–1.50)**
Pre-stroke diagnosis of dementia	**6.01 (3.47–10.42)**	**3.35 (1.53–7.32)**	**2.74 (1.32–5.70)**
Previous stroke (IS, ICH) or TIA	0.96 (0.62–1.47)	0.82 (0.45–1.48)	0.91 (0.56–1.49)

* 0.997. Bold: significant associations. Abbreviations: intracerebral haemorrhage (ICH); ischaemic stroke (IS); lacunar stroke (LACS); modified Rankin Scale (mRS); National Institute for Health Stroke Scale (NIHSS); partial anterior circulation stroke (PACS); posterior circulation stroke (POCS); total anterior circulation stroke (TACS); transient ischemic attack (TIA).

## Supplementary Materials

The following are available online at https://www.mdpi.com/2075-4418/9/3/95/s1, Table S1: Questions attempted by the partially untestable group (*n* = 58).
