# Supplementary material for: Who Is Classified as Untestable on Brief Cognitive Screens in an Acute Stroke Setting?
Source: Diagnostics (Basel). 2019 Aug 14;9(3):95. doi: 10.3390/diagnostics9030095 (PMC6787589; doi:10.3390/diagnostics9030095)
Supplement: Supplementary file 1 [file diagnostics-09-00095-s001.pdf]

Supplementary Materials

Table 1. Questions attempted by the partially untestable group (*n* = 58).

Patient	Age	Time	Date	Place	Two-person recognition	DOB	WW1	Prime minister	Months backwards	Clock draw	News item	Delayed recall	Fluency
1	y	y	y	y	y	y	y	y	y	n	y	y	y
2	y	y	y	y	y	y	y	y	n	y	y	y	y
3	y	y	y	y	y	y	y	y	n	y	n	n	n
4	y	y	y	y	y	y	y	y	y	n	y	y	y
5	y	y	y	y	y	y	y	y	y	n	y	y	y
6	y	n	n	n	n	y	n	n	n	n	n	n	n
7	y	y	y	y	y	y	y	y	n	y	y	y	y
8	y	y	y	y	y	y	y	y	y	n	n	n	n
9	y	n	y	y	n	n	n	n	n	n	n	n	n
10	y	y	y	y	y	y	y	y	y	n	n	n	n
11	y	y	y	y	y	y	n	n	n	y	n	n	n
12	y	y	y	y	y	y	y	y	y	n	n	n	n
13	y	n	n	n	n	n	n	n	n	y	n	n	n
14	y	n	y	n	n	n	n	n	n	n	n	n	n
15	y	y	y	n	n	n	n	n	n	n	n	n	n
16	y	y	y	y	y	y	y	y	y	n	n	n	n
17	y	y	y	y	y	y	n	n	n	n	n	n	n
18	y	y	y	y	y	y	y	y	y	n	n	n	n
19	y	y	y	y	y	y	y	y	y	y	y	n	n
20	y	n	y	y	n	y	n	n	n	n	n	n	n
21	y	y	y	y	y	y	y	y	y	n	y	y	y

22	y	n	n	n	n	y	n	n	n	n	n	n	n
23	y	y	y	y	y	n	n	n	n	n	n	n	n
24	y	y	y	y	y	y	y	y	y	n	y	y	y
25	y	y	y	y	y	y	n	n	n	n	n	n	n
26	y	y	y	y	y	y	y	y	y	n	y	y	y
27	y	n	y	y	n	y	n	n	n	n	n	n	n
28	y	y	y	y	n	n	n	n	n	n	n	n	n
29	y	y	y	y	y	y	y	y	y	n	n	n	n
30	n	y	n	n	n	n	n	n	n	n	n	n	n
31	y	y	y	y	y	y	y	y	y	y	y	n	n
32	y	y	y	y	y	y	y	y	y	n	y	y	y
33	y	y	n	n	n	n	n	n	n	n	n	n	n
34	y	y	y	y	y	y	y	y	y	n	y	y	y
35	y	y	y	y	y	y	y	y	y	n	y	y	y
36	y	y	y	y	y	y	y	y	y	n	y	y	y
37	y	y	y	y	y	y	y	y	y	n	y	y	y
38	y	y	y	y	y	y	y	y	y	n	y	y	y
39	y	y	y	y	y	y	y	y	y	n	n	n	n
40	y	y	y	y	y	y	y	y	y	n	y	y	y
41	y	y	y	y	y	y	y	y	y	n	y	y	y
42	y	n	n	y	n	y	n	n	n	n	n	n	n
43	y	y	y	y	y	y	y	y	y	n	y	y	y
44	y	y	n	n	n	n	n	n	n	n	n	n	n
45	y	y	y	y	y	y	y	y	y	n	y	y	y
46	y	y	y	y	y	y	n	n	n	n	n	n	n
47	y	y	y	y	y	y	y	y	y	n	n	n	n
48	y	y	y	y	y	y	y	y	y	n	y	y	y
49	y	y	y	y	y	y	y	y	y	n	y	y	y
50	y	y	y	y	y	y	y	y	y	n	y	y	y

51	y	y	y	y	y	y	y	y	y	n	y	y	y
52	y	y	y	y	y	y	y	n	n	n	n	n	n
53	y	y	y	n	n	n	n	n	n	n	n	n	n
54	y	y	y	y	y	y	y	y	y	n	y	y	y
55	y	y	y	y	y	y	y	y	y	n	y	y	y
56	y	y	y	y	y	y	y	y	y	n	y	y	y
57	y	y	y	y	y	y	y	y	y	n	y	y	y
58	y	y	y	y	y	y	y	y	y	n	y	y	y
Y totals	57	50	51	49	44	48	39	38	35	7	29	27	27
